# AIDS and HIV Infection after Thirty Years

**DOI:** 10.1155/2013/731983

**Published:** 2013-01-10

**Authors:** Giuseppe Ippolito, Jay A. Levy, Anders Sonnerborg, Ferdinand Mugusi, Ferdinando Dianzani

**Affiliations:** ^1^Department of Medicine, AIDS Research Institute, National Institute for Infectious Diseases Lazzaro Spallanzani, Via Portuense, 292-00149 Rome, Italy; ^2^AIDS Research Institute, University of California at San Francisco, San Francisco, CA, USA; ^3^Unit of Infectious Diseases, Department of Medicine, Karolinska Institutet, Stockholm, Sweden; ^4^Department of Internal Medicine, Muhimbili University of Health and Allied Sciences (MUHAS), Dar es Salaam, Tanzania; ^5^Department of Virology, National Institute for Infectious Diseases Lazzaro Spallanzani, Via Portuense, 292-00149 Rome, Italy; ^6^Sapienza University of Rome, Piazzale Aldo Moro, 5-00185 Rome, Italy

Acquired Immunodeficiency Syndrome (AIDS), caused by the Human Immunodeficiency virus (HIV), first appeared in Western countries in 1981 as a disease leading to a high death rate. It initially approached 100% after 5–10 years of the diagnosis. As evidenced from articles in this special issue, much progress has been made in the global fight against HIV/AIDS. Valuable information has been gathered on the virus and the host immune response to this new human pathogen. Successful therapies have been developed and approaches to control HIV spread have been instituted. The epidemic, however, continues to affect the Western countries and, importantly, the international community in resource-limited countries. According the United Nations Programme on HIV/AIDS (UNAIDS) estimates, published for the World AIDS Day 2012, 34 million (31.4–35.9) people are living with HIV, 2.5 (2.1–2.8) million people became new infected with HIV, and 1.7 (1.5–1.9) million died from AIDS-related illnesses. (UNAIDS Global report: UNAIDS report on the global AIDS epidemic 2012. UNAIDS/JC2417E available at http://www.unaids.org/en/resources/campaigns/20121120_globalreport2012/).

In the last 30 years since the recognition of the disease, clinicians, scientists, public health officials, policy makers, and volunteers have worked together to take care of people with HIV infection, to identify new drugs and evaluate their efficacy, to better understand the virus and to assess its pathogenicity, to find new tools to diagnose and prevent HIV infection, to assess the role of coinfections, and to mitigate the adverse events from antiretroviral treatment. Essentially, they have focussed on directions to manage patients according to a model of clinical excellence.

 This large investment of resources has given impressive results and represents a welcomed scientific success. As noted in this issue, after 3 decades, AIDS has become at least for many industrialized countries a chronic disease affording patients a near-normal lifestyle and survival, if adequately treated [1, 2]. This year, UNAIDS reported an unprecedented acceleration in the clinical response to HIV/AIDS with more than a 50% reduction in the rate of new HIV infections even in some low- and middle-income countries. Impressive results have also been reached in resource-limited countries in terms of access to treatment. According to the above-mentioned UNAIDS Global report, worldwide the number of people with access to antiretroviral therapy has increased by 63% in the last 24 months. In sub-Saharan Africa the number of people on antiretroviral treatment has increased by 59% in the last two years and the number of AIDS-related deaths has been reduced by one-third in the last six years. Nevertheless, the clinical spectrum today, even with therapy, can include cardiovascular disease [3], infectious and noninfectious cancers [4], osteopenia/osteoporosis [5], liver and renal disease [6], and neurocognitive decline [7].

Importantly, the treatments for HIV/AIDS are very expensive and difficult to afford in resource-limited countries. Even in high-income countries, drug expenditure for treatment of HIV/AIDS today represent, according to recent estimates, 62,4% of total cost versus a mean of 20% for other chronic diseases and the cost per person treated is 38% higher than the treatment of cancer. 

Global economic value of DALYs by HIV/AIDS is 21.6% of the value for cancer and 26% of heart diseases [8]. Concern over the costs of health care is universal and experience gained from studies of HIV/AIDS could help to influence the model of access to care for other clinical conditions. 

For this special issue, a policy to have papers from researchers from resource-limited countries was adopted. The articles chosen cover several areas of research in different countries. In the paper by M. Harris et al., the problem of cost-effectiveness of highly active antiretroviral therapy for Multidrug-Resistant (MDR) HIV has been evaluated. The authors consider the possibility for patients with MDR to achieve full and durable viral suppression along with the cost of different options for treatment. The proposed solution could be a basis for careful planning and strategic use of antiretroviral drugs.

In A. E. Horace review, the evolution of the pharmacists' role in treatment of HIV-infected patients is presented. Facilitation of access to treatment of HIV-infected patients is discussed with attention to increasing effectiveness and adherence to prescribed medicines. In addition, measuring the CD4+ cell count and viral load revolutionized the possibility for monitoring the evolution of infection in HIV-infected persons. CD4+ cell counts represent the first successful application on a large scale of this technique in western and African countries. In A. Akinbami et al. paper, the authors present an evaluation, based on CD4+ cell count, of the percentage of HIV-infected people who require antiretroviral therapy at enrollment in an HIV treatment and care center in Nigeria.

Neurological manifestations, including HIV-dementia and HIV-associated neurocognitive disorder (HAND), are among the most threatening complications of HIV infection [7]. Although their incidence has decreased among people who have access to combination antiretroviral treatments, new neurological problems are being observed in countries where treatments are available. They are related to both changes in the natural history of old diseases as well as emergence of novel entities. 

Two contributions on this topic are presented in this issue. In O. O. Oshinaike et al. report, the performance of the minimental state examination (MMSE) and international HIV dementia scale (IHDS) in assessing neurocognitive function in treated HIV/AIDS has been compared in Nigeria. In the second paper, M. L. Giancola et al. investigate the effect of antiretroviral therapy and single antiretroviral drugs on the cerebrospinal fluid HIV-RNA levels in HIV-infected patients affected by neurological disorders enrolled in a large multicentric Italian cohort.

Oral lesions and other oral manifestations are frequent in persons with HIV infection. Candidiasis is a common finding and lesions of the oral cavity occur in about one-third of patients with AIDS-associated Kaposi's Sarcoma (KS). Two clinical studies on this topic by the same group in South Africa appear in this issue. The first characterizes the features of oral HIV-KS and describes the patterns of evolution of the disease [9]. The second compares the prevalence and the rate of progression of chronic periodontitis in HIV-positive and -negative subjects [10].

Today the efficacy of antiretroviral drugs and the impressive changes in the progression of the HIV disease allow mothers perinatally infected to have almost a universal prevention in transmitting HIV infection to their offspring [11]. In this regard, M. L. Badell and M. Lindsay review various aspects related to maternal, obstetrical, and neonatal risks associated with pregnancy in perinatally HIV-infected mothers.

The determinants of the different time course for progression of HIV infection is still not clearly defined. J. C. Gaardbo et al. describe immunological features of controllers and long-term nonprogressors where knowledge on their ability to control HIV infection could lead to the development of immune therapies or a therapeutic vaccine.

With the introduction of antiviral therapy, deaths in HIV-infected people have been dramatically reduced with an improved quality of life for treated patients [12]. In this regard, A. Stewart et al. investigated the changes that occur in the care of persons living with HIV in a hospice.

These papers published in this special issue present exciting, insightful observations on the state of HIV/AIDS and the emergence of future topics of research for investigators. In this rapidly developing field of study, interdisciplinary and challenging research, performed in both industrialized and resource-limited countries, can bring critically important information for the life and social sciences, for public health, and for overall health care. Development of new preventive and therapeutic strategies is being encouraged as well as new models of health care. The aim of this special issue is to contribute to the development of knowledge on HIV/AIDS, to recognize the great progress many facets of medicine have made towards its complete control, and to recognize the challenges that still remain.
